# Unraveling Fibrous Tonsil Lesions: A Case Series on Diagnostic Dilemmas in Pediatric Tonsillectomy

**DOI:** 10.1155/crot/5486047

**Published:** 2026-04-03

**Authors:** Isabel Snee, Daniel Swanson, Kelly Scriven, Earl Harley

**Affiliations:** ^1^ Georgetown University Medical Center, Washington, DC, USA, georgetown.edu; ^2^ Department of Otolaryngology-Head and Neck Surgery, MedStar Georgetown University Hospital, Washington, DC, USA, medstargeorgetown.org

**Keywords:** fibrous tonsil, otolaryngology, pediatric otolaryngology, tonsillectomy and adenoidectomy

## Abstract

**Purpose:**

This study examines the challenges associated with diagnosing and managing fibrous tonsillar lesions in pediatric patients undergoing tonsillectomy and adenoidectomy. In this report, we outline our strategy for navigating these patients’ clinical presentation while maintaining a broad differential diagnosis in pediatric tonsil lesions.

**Methods:**

This retrospective case series analyzes two pediatric patients who underwent tonsillectomy and adenoidectomy for symptoms related to sleep‐disordered breathing and obstructive sleep apnea.

**Results:**

Both cases displayed substantial tonsillar hypertrophy with fibrous, polyploid lesions. Subsequent histopathological analysis revealed fibroepithelial polyps (FEPs) in one case, while the other demonstrated tonsillar hyperplasia. Following surgery, recovery for both cases was largely unremarkable, with one patient developing a transient fever and the other managing mild postoperative pain with analgesics.

**Conclusions:**

These cases illustrate the diagnostic complexity of pediatric tonsillar pathology and emphasize the need to maintain a broad differential diagnosis for tonsillar lesions. Thorough histopathological evaluation and transparent communication with patients and their families are essential for enhancing patient‐centered care.

## 1. Introduction

Fibroepithelial polyps (FEPs) are rare, benign lesions characterized by polypoid overgrowth of fibrous connective tissue covered by squamous epithelium [1–3]. They are most reported in cutaneous and oral sites, with only limited documentation involving the palatine tonsil [4–6]. Although FEPs are considered benign with minimal malignant potential, their irregular, fibrous, or nodular appearance may clinically mimic neoplastic processes [7, 8].

Tonsillar FEPs are particularly uncommon in the pediatric population [9]. When atypical tonsillar lesions are encountered in children, the differential diagnosis is broad and includes infectious, inflammatory, and malignant etiologies [7, 8]. Given the potential for serious pathology, including lymphoma, histopathologic evaluation remains essential in distinguishing benign lesions from malignancy.

In this case series, we present two pediatric patients who underwent tonsillectomy and adenoidectomy for sleep‐disordered breathing and were found intraoperatively to have fibrous, polypoid tonsillar lesions concerning for neoplasia. The objective of this report is to highlight the rarity and diagnostic complexity of FEPs in children, emphasize the importance of maintaining a broad differential diagnosis, and underscore the role of histopathologic evaluation and clear perioperative communication in optimizing patient care.

## 2. Case Presentation

### 2.1. Case One

A 4‐year‐old male with seasonal allergies and a history of neonatal respiratory distress presented with a 1.5‐year history of snoring and restless sleep without witnessed apneic episodes. Medical management with cetirizine and fluticasone provided minimal improvement. Physical examination revealed 3+ bilateral tonsillar hypertrophy and inferior turbinate hypertrophy. Polysomnography demonstrated severe obstructive sleep apnea with an apnea–hypopnea index (AHI) of 14.2 and an oxygen nadir of 82%. Tonsillectomy with turbinate reduction was recommended.

**FIGURE 1:**
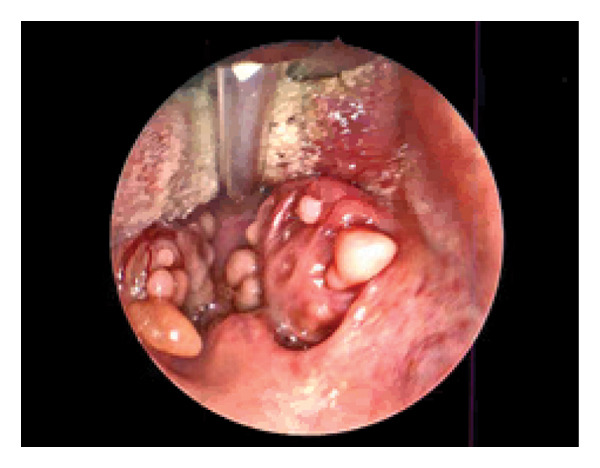
Case 1 view of an FEP inside the patient’s mouth.

Intraoperatively, the tonsils were markedly enlarged (3‐4+) with diffuse, fibrous, polypoid lesions covering both surfaces (Figure 1). The unusual appearance raised concern for possible neoplastic pathology. An extracapsular tonsillectomy was performed, and representative tissue was sent for pathologic evaluation.

Histopathologic analysis revealed polypoid squamous mucosa with underlying fibrosis, chronic inflammation, and reactive lymphoid follicles, consistent with FEP. No dysplasia or malignancy was identified.

Postoperatively, the patient developed a transient fever to 103°F lasting 3‐4 days without the need for antibiotics. Recovery was otherwise uneventful, and no additional intervention was required.

### 2.2. Case Two

An 11‐year‐old male with a history of PTEN mutation, autism spectrum disorder, asthma, and allergic rhinitis presented with chronic nasal obstruction, congestion, and snoring. Medical therapy provided limited symptom relief. Examination revealed 3+ bilateral tonsillar hypertrophy, and imaging demonstrated significant adenoid hypertrophy. The family elected to proceed with adenotonsillectomy.

Intraoperatively, the tonsils appeared firm and nodular with multiple frond‐like projections extending beyond the tonsillar capsule (Figure 2). Given the atypical and indurated appearance, the procedure was converted from intracapsular to extracapsular tonsillectomy following discussion with the patient’s mother.

FIGURE 2Case 2 view of an FEP inside the patient’s mouth (a) and the specimen sent to pathology for further pathology workup (b).

**Figure:**
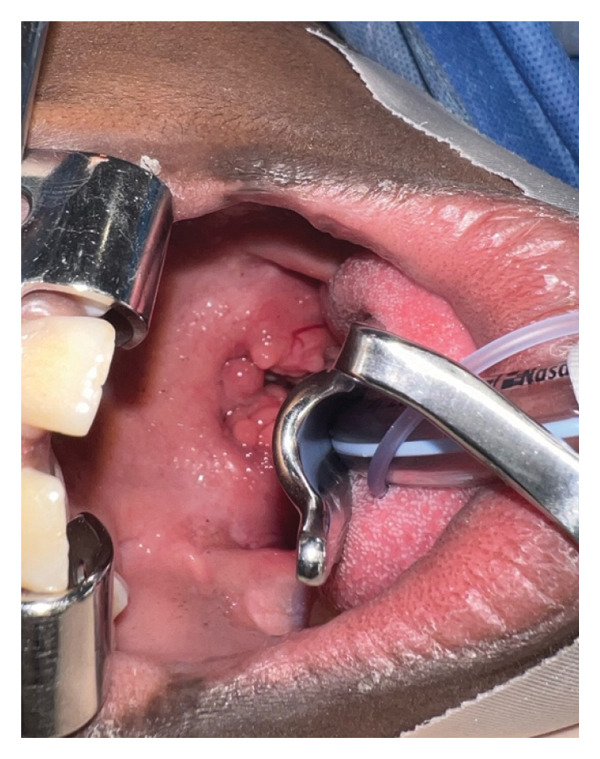
(a)

**Figure:**
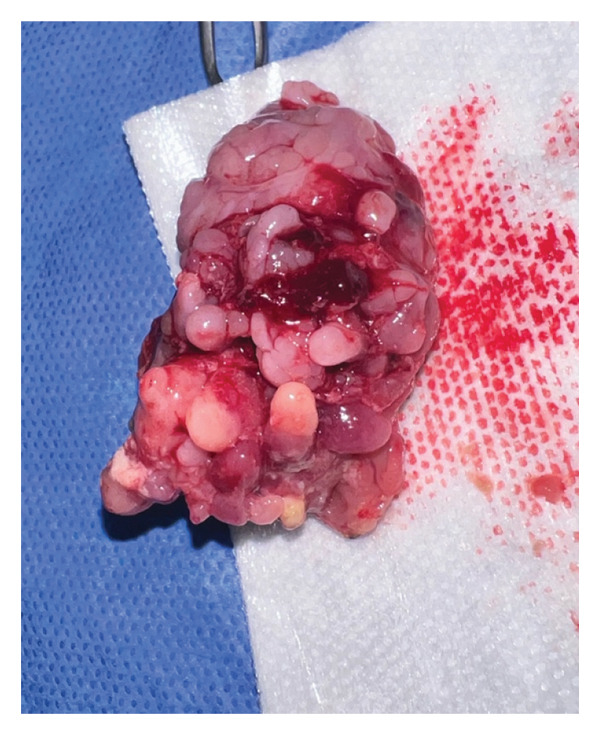
(b)

Histopathology demonstrated reactive follicular changes consistent with benign reactive lymphoid hyperplasia. The specimen was negative for lymphoma or other malignancy.

Postoperative recovery was notable for transient pain managed with oral analgesics. No further complications occurred.

## 3. Discussion

This case series highlights two distinct but clinically overlapping presentations of atypical tonsillar pathology in pediatric patients undergoing surgery for sleep‐disordered breathing. In the first case, diffuse fibrous and polypoid tonsillar lesions were ultimately diagnosed as an FEP, a rare benign entity in children. In the second case, firm and nodular tonsillar enlargement raised intraoperative concern for malignancy but was found to represent benign reactive lymphoid hyperplasia. Together, these cases underscore the diagnostic uncertainty that may arise when tonsillar morphology deviates from expected hypertrophy.

FEPs are benign lesions characterized by fibrous stromal overgrowth covered by squamous epithelium. Although most described in cutaneous and oral locations, tonsillar involvement is rare, particularly in pediatric patients. Reported cases suggest a mild male predominance and greater prevalence in older populations [1–3]. Pediatric tonsillar FEPs remain sparsely documented in the literature, and few reports describe their intraoperative appearance in the setting of sleep‐disordered breathing. By presenting two distinct intraoperative presentations, one confirmed FEP and one benign reactive tonsillar hyperplasia, this series expands awareness of how fibrous or nodular tonsillar morphology may present and reinforces the need for careful diagnostic evaluation.

This case report illustrates how benign reactive lymphoid hyperplasia may clinically mimic malignancy when tonsillar tissue appears indurated or nodular. Since lymphoma remains the most common extranodal head and neck malignancy in children, it must be considered in the differential diagnosis of atypical tonsillar lesions. Although pediatric tonsillar malignancy is rare, the reported incidence is approximately 0.021 per 100,000 annually, with non‐Hodgkin’s lymphoma accounting for most cases [10]. Age‐specific incidence increases from approximately 5.9 per million in children under five years of age to 15 per million in adolescents [9]. These epidemiologic data reinforce that abnormal tonsillar morphology warrants histopathologic confirmation rather than reliance on clinical impression alone. Clinical presentation of tonsillar lymphoma may vary by age. A systematic review suggests that snoring may predominate in younger children, lymphadenopathy in school‐aged patients, and dysphagia in adolescents [11]. Tonsillar asymmetry has also been reported as a common presenting feature in pediatric lymphoma [11–13]. Notably, neither of our patients demonstrated tonsillar asymmetry or significant cervical lymphadenopathy, findings that may help contextualize operative concern. However, the absence of classic warning signs does not eliminate the need for definitive histopathologic assessment.

From a clinical management perspective, these cases emphasize that atypical tonsillar texture, firmness, or polypoid morphology should prompt thoughtful surgical decision‐making and routine submission of specimens for pathologic evaluation. Histopathology remains the gold standard for distinguishing benign proliferative processes from malignancy. Reliance solely on intraoperative appearance may be misleading, as demonstrated by the second case.

An important applied implication highlighted here is perioperative communication. Intraoperative concern for malignancy in the second case prompted conversion from intracapsular to extracapsular tonsillectomy following discussion with the patient’s family. Transparent communication facilitated shared decision‐making despite diagnostic uncertainty. When atypical findings arise intraoperatively, a structured approach to family communication may be beneficial. This includes clearly describing the unexpected finding in accessible language and explaining the differential diagnosis and associated uncertainty. Once this foundation has been established, clinicians should outline the rationale for any modification in surgical approach and set expectations regarding pending histopathologic results and potential next steps. Such an approach helps preserve trust, reduce anxiety, and reinforce patient‐centered care during situations of clinical ambiguity.

Collectively, this case series contributes to the limited pediatric literature on FEPs of the tonsil and illustrates how both rare benign lesions and reactive lymphoid processes may mimic malignancy. These cases reinforce three key principles: maintaining a broad differential diagnosis, routinely confirming diagnosis through histopathologic evaluation, and prioritizing clear communication with families when unexpected findings arise. Recognition of these uncommon presentations may improve diagnostic confidence and surgical decision‐making in pediatric otolaryngology.

An additional limitation of this report is the absence of representative histopathologic images. While intraoral and gross specimen photographs are included, histologic slides would further enhance visualization of the fibroepithelial architecture and reactive lymphoid features described. These images were not available at the time of manuscript preparation.

## 4. Conclusion

These cases highlight the diagnostic challenges posed by atypical tonsillar lesions in pediatric patients. Although FEPs are rare and benign, their intraoperative appearance may raise concern for malignancy. Clinicians should maintain a broad differential diagnosis when encountering unusual tonsillar findings, pursue definitive histopathologic evaluation to exclude serious pathology, and prioritize clear perioperative communication with patients and their families. Recognition of this uncommon entity may help guide appropriate surgical decision‐making and optimize patient‐centered care.

## Funding

This research did not receive any specific grant from funding agencies in the public, commercial, or not‐for‐profit sectors.

## Ethics Statement

Written informed consent was obtained from the patients’ legal guardians for publication of this case series and accompanying images. Identifying information has been omitted to protect patient confidentiality.

## Conflicts of Interest

The authors declare no conflicts of interest.

## Data Availability

The data that support the findings of this study are available upon request from the corresponding author. The data are not publicly available due to privacy or ethical restrictions.
